# Mitochondria and sensory processing in inflammatory and neuropathic pain

**DOI:** 10.3389/fpain.2022.1013577

**Published:** 2022-10-17

**Authors:** P. Silva Santos Ribeiro, Hanneke L. D. M. Willemen, Niels Eijkelkamp

**Affiliations:** Center for Translational Immunology, University Medical Center Utrecht, Utrecht University, Utrecht, Netherlands

**Keywords:** mitochondria, sensory neurons, inflammation, neuro-inflammation, chronic pain, rheumatic disease, neuropathy

## Abstract

Rheumatic diseases, such as osteoarthritis and rheumatoid arthritis, affect over 750 million people worldwide and contribute to approximately 40% of chronic pain cases. Inflammation and tissue damage contribute to pain in rheumatic diseases, but pain often persists even when inflammation/damage is resolved. Mechanisms that cause this persistent pain are still unclear. Mitochondria are essential for a myriad of cellular processes and regulate neuronal functions. Mitochondrial dysfunction has been implicated in multiple neurological disorders, but its role in sensory processing and pain in rheumatic diseases is relatively unexplored. This review provides a comprehensive understanding of how mitochondrial dysfunction connects inflammation and damage-associated pathways to neuronal sensitization and persistent pain. To provide an overall framework on how mitochondria control pain, we explored recent evidence in inflammatory and neuropathic pain conditions. Mitochondria have intrinsic quality control mechanisms to prevent functional deficits and cellular damage. We will discuss the link between neuronal activity, mitochondrial dysfunction and chronic pain. Lastly, pharmacological strategies aimed at reestablishing mitochondrial functions or boosting mitochondrial dynamics as therapeutic interventions for chronic pain are discussed. The evidence presented in this review shows that mitochondria dysfunction may play a role in rheumatic pain. The dysfunction is not restricted to neuronal cells in the peripheral and central nervous system, but also includes blood cells and cells at the joint level that may affect pain pathways indirectly. Pre-clinical and clinical data suggest that modulation of mitochondrial functions can be used to attenuate or eliminate pain, which could be beneficial for multiple rheumatic diseases.

## Introduction

Rheumatic diseases are often grouped under the term “arthritis”, which is used to describe over 100 diseases that include rheumatoid arthritis (RA), osteoarthritis (OA), fibromyalgia, systemic lupus erythematosus (SLE), ankylosing spondylitis (AS), psoriatic arthritis (PsA), and juvenile idiopathic arthritis (JIA). These rheumatic diseases are characterized by inflammation and tissue damage (1). In several of these rheumatic pathologies, e.g., RA and SLE, an autoimmune component is present (2). In most rheumatic diseases, joints, cartilage, tendons, ligaments, bones, and muscles are the main and most commonly affected tissues. Although specific mechanisms differ between diseases, the release of pro-inflammatory molecules or damage-associated molecules by cells, contributing to tissue inflammation and destruction, is a common feature among these rheumatic conditions. For example, in RA, fibroblast-like synoviocytes (FLSs) release pro-inflammatory molecules, leading to tissue damage. In OA, chondrocyte cell death triggers the release of inflammatory cytokines in surrounding tissues (3). Another shared element among rheumatic patients is that they often report pain as their most debilitating symptom (4–7). Fibromyalgia has an unknown pathophysiology, but is characterized by chronic widespread pain, which is also present in 65%–80% of SLE patients (8).

Although inflammatory components are present in rheumatic diseases, it is still not completely understood what drives pain in these diseases. The magnitude of inflammation in RA or SLE, or the severity of damage assessed by radiographic knee damage in OA, do not correlate with pain intensity (8–13). Moreover, 12%–70% of RA patients have persistent pain after remission or under minimal disease activity (13–15). In OA, 10%–40% of the patients still have pain even 5 years after total knee replacement surgery (11). In summary, pain is not directly associated with the magnitude of damage/inflammation and often persists even when the inflammation or damage is minimal or resolved.

Chronic pain affects at least 20% of the world population (∼1.4 billion people), with rheumatic diseases, such as OA and RA, contributing to approximately 40% of these chronic pain cases (1, 16–19). Currently available pain treatments include analgesics [e.g., paracetamol, non-steroidal anti-inflammatory drugs (NSAIDs), opioids, and steroids], physiotherapy or surgery (20). The available treatments are often not very effective to treat chronic pain (21, 22). NSAIDs and steroids are the most common treatments for rheumatic diseases, due to their anti-inflammatory and analgesic properties. Nonetheless, renal, hepatic, cardiovascular and gastrointestinal adverse effects are commonly reported (23, 24). Most importantly, despite their anti-inflammatory properties, the highest pain reduction reported was lower than 10% in average, further supporting a lack of correlation between inflammation and pain intensity in rheumatic disease (24). Additionally, preclinical evidence supports clinical observations, as the anti-inflammatory corticosteroid (dexamethasone) prevents acute inflammatory pain, but no longer has an effect when pain becomes chronic (25). Finally, opioids, the last resource in terms of analgesics, reduce pain intensity 20%–30% in OA patients and only 10% in musculoskeletal pain in general (22). The limited therapeutic outcome of opioids comes with potential side effects, including risk for addiction, which significantly contributes to the current opioid crisis (26, 27).

Most studies on pain in rheumatic disease focus on cellular and molecular alterations in joint tissues induced by inflammation. It is well known that inflammatory mediators can directly activate or sensitize sensory neurons (28–30). Yet, given that pain often persists with minimal or no joint inflammation/damage, it is likely that changes in the peripheral and central nervous system also drive pain in rheumatic disease. Indeed, inflammation and tissue damage may determine long lasting neuronal plasticity (28–30). Joint inflammation alters protein expression in sensory neurons that innervate the joints and have their cell bodies in the dorsal root ganglia (DRG) (31). In patients with mild and severe OA, sensory innervation is increased in the subchondral bone (32), potentially enhancing pain signaling pathways. Primary sensory neurons synapse in the dorsal horn in the spinal cord. In rheumatic diseases, exacerbated sensory neuron activity or neuronal damage may promote the activation of astrocytes and microglia in these spinal regions (31). Increased activation of glia may explain why the levels of pro-inflammatory cytokine interleukine-1β (IL-1β) are increased in the cerebrospinal fluid of RA patients (33). Overall, the magnitude of rheumatic pain likely depends on the interplay between joint damage and pain signaling pathways at various levels including DRG, spinal cord and brain.

Mitochondria are essential for a myriad of cellular processes and play a key role in regulating inflammatory responses (34), but also neuronal functions. Neurons have a higher energetic demand in comparison to other cell types (35). Mitochondria are a main source of adenosine triphosphate (ATP) in neurons. This ATP is essential to maintain the membrane potential and restore it after an action potential (36). Mitochondria are Ca^2+^ reservoirs, regulating intracellular Ca^2+^ concentration (37, 38). Moreover, they are a source of reactive oxygen species (ROS). Mitochondria control the release of neurotransmitters, neuronal excitability, signaling and plasticity (39). The role of mitochondria in neuronal activity has been extensively explored in the context of neurodegenerative diseases. For example, changes in mitochondrial axonal transport or the removal of damaged mitochondria in neurons contribute to Alzheimer's disease (40) and Parkinson's disease, respectively (41). Furthermore, intercellular transfer of mitochondria from astrocytes or macrophages to neurons promotes neuronal survival after stroke, or resolves inflammatory pain, respectively (25, 42). However, the role of mitochondria in the regulation of pain is only begun to be understood. Importantly, modulating mitochondrial functions in sensory neurons reduces hyperalgesia in pre-clinical models of neuropathic and inflammatory pain (43–48). Genetic disruption of complex IV of the mitochondrial electron transport chain in primary sensory neurons causes pain hypersensitivity (49). Moreover, 70% of humans with inherited mitochondrial deficits develop chronic pain (50). Thus these data suggest an association between mitochondrial dysfunction, neuronal activity, and chronic pain.

The role of mitochondria in rheumatic diseases in general has been discussed in other reviews (51–55). However, their role in the development of rheumatic pain has not been well covered. In this review, we will focus on the contribution of mitochondria to pain in rheumatic diseases. In the following sections, we will discuss how mitochondria affect sensory processing and pain development, using inflammatory and neuropathic pain models. Furthermore, we discuss to what extent modulating mitochondrial functions may be promising to treat chronic rheumatic pain.

## Mitochondrial dysfunction in the nervous system and pain

Inflammatory pain is caused by tissue damage and inflammatory responses, while neuropathic pain is usually described as a consequence of neuronal damage in the peripheral or central nervous system (16, 56, 57). Pain in rheumatic diseases is often considered as inflammatory, but nerve damage may also contribute to pain. Vice versa, neuropathic pain shares features of inflammatory pain. Inflammation can trigger neuronal damage and consequently neuropathic pain. Therefore, the discrimination between these two types of pain can be sometimes difficult (16, 56). As example, inflammatory mediators sensitize afferent nociceptive nerve fibers and trigger damage of sympathetic nerve fibers in the joints of rodents in experimental models of inflammatory arthritic pain (58). In addition, several OA patients show signs of neuropathic pain (9). Therefore, we will discuss findings in common inflammatory pain models [e.g., induced by carrageenan or Complete Freund's Adjuvant (CFA)], but also discuss the role of mitochondria in neuropathic pain (e.g., induced by nerve ligation or chemotherapeutic drugs), as it may inform us on what happens in rheumatic diseases when nerves are damaged. These models are very relevant and their significance in the pain field is discussed elsewhere (59, 60).

**Figure 1:**
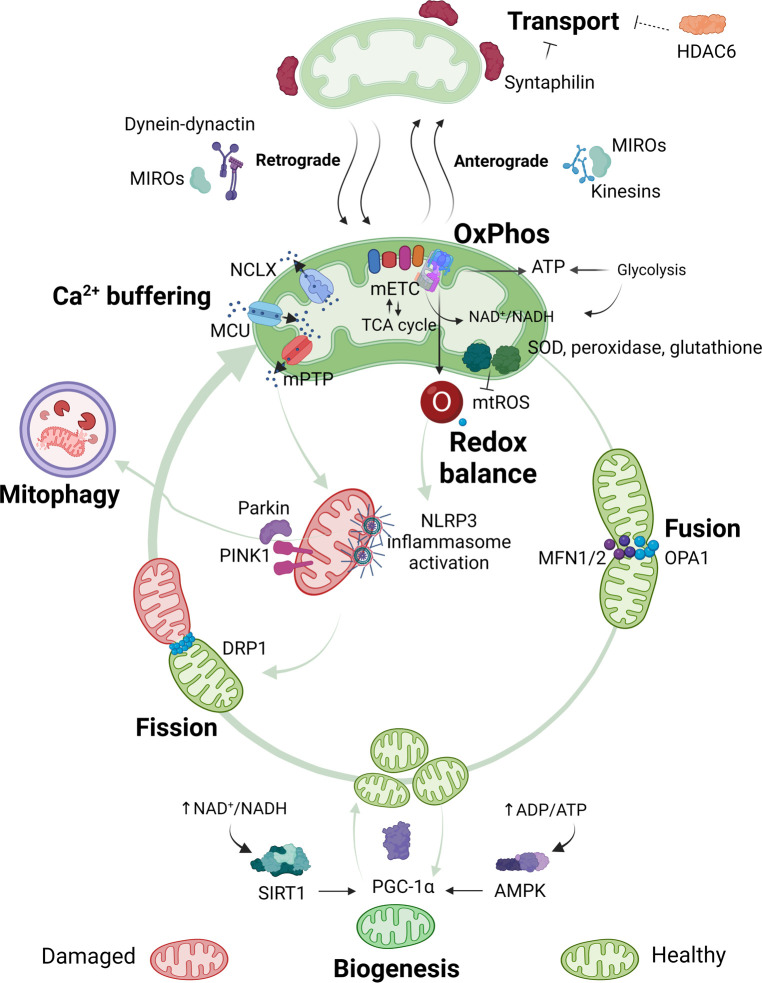
Overview of mitochondrial functions. Mitochondria are pleiotropic organelles with multiple functions. The most relevant ones are depicted in the figure. Mitochondria produce ATP *via* oxidative phosphorylation (OxPhos), which involves the interaction between the mitochondrial electron transport chain (mETC) and enzymes from the tricarboxylic acid (TCA) cycle. The mETC is the main source of mtROS, which are eliminated by mitochondrial antioxidant enzymes like superoxide dismutase (SOD), peroxidase or glutathione. Ca^2+^ enters the mitochondria through the mitochondrial Ca^2+^ uniporter (MCU) and is released through the Na^+^/Ca^2+^ exchanger (NCLX), or in case of Ca^2+^ overload in the mitochondrial matrix through assembly of the mitochondrial permeability transition pore (mPTP). Increased mtROS production or mitochondrial Ca^2+^ concentration can trigger nucleotide-binding oligomerization domain-like receptor pyrin domain containing 3 (NLRP3) inflammasome activation and/or binding of Parkin and PTEN-induced kinase 1 (PINK1) to mitochondria, which leads to the elimination of damaged mitochondria *via* mitophagy. Quality control mechanisms, such as mitophagy, fusion, fission and biogenesis ensure a healthy and functional mitochondria pool. Optic atrophy 1 (OPA1) and mitofusins1 and 2 (MFN1 or MFN2) allow mitochondria to merge (fusion), while dynamin-related protein (DRP1) permits mitochondria to segment (fission). New mitochondria are produced through biogenesis, which is promoted by peroxisome proliferator-activated receptor gamma coactivator 1-alpha (PGC-1α). Decreased energy production detected by energy sensors sirtuin1 (SIRT1) and adenosine monophosphate (AMP)-activated protein kinase (AMPK) will activate PGC-1α. Quality control mechanisms are highly dynamic and adapt to the cells' needs. Mitochondria are transported to the area where they are required. In neurons, mitochondria travel from the soma to the axons and damaged mitochondria back to the soma. Anterograde transport of mitochondria is mediated by mitochondrial Rho GTPases (MIROs) and kinesins, whilst retrograde transport is mediated by MIROs and dynein-dynactin complexes. Syntaphilin is an anchor protein that stabilizes mitochondria in a fixed spot. Histone deacetylase 6 (HDAC6) indirectly reduces mitochondrial transport by deacetylation of the cytoskeletal α-tubulin, a protein that facilitates dynein-dependent mitochondrial transport when acetylated. Figure created with BioRender.com.

Mitochondria are presumably developed from engulfed prokaryotes that were once independent organisms (61). Mitochondria are complex organelles that have their own mitochondrial deoxyribonucleic acid (mtDNA) and a very characteristic morphology. Each mitochondrion is formed by a double membrane. The outer membrane has a composition similar to the plasma membrane of an eukaryotic cell. The inner membrane is organized in several cristae to maximize efficient ATP production during oxidative phosphorylation (OxPhos) (53, 62). The main mitochondrial functions and associated pathways are displayed in Figure 1. Each of these functions will be discussed in the context of rheumatic pain. A detailed overview of all specific alterations in mitochondrial functions reported in pre-clinical studies and in humans with rheumatic diseases can be found in Tables 1, 2, respectively.

**Table 1:** Detailed overview of pre-clinical findings showing mitochondrial dysfunction in inflammatory and neuropathic pain models.

Mitochondrial function	Protein/pathway affected	Pathology	Tissue/cell type	Species	References
Mitochondrial respiration	↓ OxPhos	Transient inflammatory pain (carrageenan)	DRG neurons	Mice	(25)
	↑ OxPhos and TCA cycle	Transient inflammatory pain (PGE_2_)	DRG neurons	Mice	(63)
	↑ OxPhos	Hyperalgesic priming (PGE_2_ + carrageenan)	DRG neurons	Mice	(64)
	↓ OxPhos	Chronic inflammatory/RA pain (CFA)	Spinal cord	Rats	(65)
	↓ Expression of mETC related proteins		DRG	Mice	(66)
	↑ ATPSc-KMT expression		DRG neurons	Mice	(46)
	↓ ATP levels	OA (meniscal destabilization)	Chondrocytes	Rabbits	(67)
		OA (spontaneous)	Chondrocytes	Hartley guinea pigs	(68)
	↓ OxPhos	CCI induced neuropathic pain	Spinal cord	Mice	(65)
Oxidative stress	↑ MtROS	Transient inflammatory pain (carrageenan)	Spinal cord	Mice	(69)
	↑ MtROS	SNL induced neuropathic pain	Spinal cord neurons	Rats	(70)
		Cisplatin induced CINP	DRG neurons	Mice	(71)
Ca^2+^ buffering	↑ Intracellular Ca^2+^	Transient inflammatory pain (carrageenan)	Brain neurons	Rats	(72)
	↑ Evoked Ca^2+^ transients	Chronic inflammatory/RA pain (CFA)	DRG neurons	Rats	(73, 74)
	↓ Mitochondrial Ca^2+^ storage capacity	SNL induced neuropathic pain	DRG neurons	Rats	(75)
	↑ mPTP-mediated Cytochrome C release	SNI induced neuropathic pain	Spinal cord neurons	Rats	(76, 77)
	↑ Mitochondrial Ca^2+^ uptake	Paclitaxel induced CINP	Skin innervating DRG neurons	Rats	(78)
	↑ MCU-mediated mitochondrial Ca^2+^ uptake	Painful diabetic neuropathy (high fat diet)	DRG neurons	Mice	(79)
Mitophagy	↑ PINK1 and Parkin expression	OA (MIA)	Knee cartilage	Rats	(44)
	↑ PINK1 expression	SNL induced neuropathic pain	Spinal cord neurons	Rats	(80)
Mitochondrial biogenesis	↓ AMPK activity, SIRT-1, PGC-1α, TFAM, and NRF-2 expression	OA (medial meniscectomy, spontaneous due to aging)	Chondrocytes	Mice	(81, 82)
Fusion/fission	↑ DRP1 expression and ↓ OPA1 expression	Chronic inflammatory/RA pain (CFA)	Spinal cord	Rats	(83)
	↑ DRP1 expression and ↓ OPA1 expression	CIBP (intramedullary injection of mammary carcinoma cells in the tibia)	Spinal cord	Rats	(84)
Mitochondrial transport	↑ HDAC6 activity	Cisplatin induced CINP	DRG neurons, tibial nerve	Mice	(85, 86)
NLRP3 inflammasome activation	↑ NLRP3 and IL-1β expression	Chronic inflammatory/RA pain (CFA)	Spinal cord	Mice	(87)
	↑ NLRP3 expression	Experimental arthritis/RA pain (CIA)	Synovial tissues	Mice	(88)
	↑ NLRP3, Caspase-1, ASC, IL-1β, IL-18 expression	OA (MIA)	Synovial tissues, FLSs	Rats	(89, 90)
	↑ NLRP3, Caspase-1, ASC expression	OA (destabilization of the medial meniscus)	Cartilage	Rats	(91)
	↑ NLRP3, ASC, Caspase-1, IL-1β, IL-18	CCI induced neuropathic pain	Spinal cord astrocytes and microglia, spinal cord, DRG, sciatic nerves	Mice, rats	(92–95)
	↑ NLRP3, ASC, P-Caspase-1, Caspase-1, IL-1β expression	SNL induced neuropathic pain	Spinal cord	Mice	(96)
	↑ NLRP3, ASC, IL-1β and IL-18 expression	CIBP (injection of mammary carcinoma cells in the tibia)	Spinal cord neurons	Rats	(97)
	↑ NLRP3 and ASC expression, increased IL-1β release	Oxaliplatin induced CINP	Spinal cord	Rats	(98)
	↑ NLRP3 expression	Bortezomib induced CINP	DRG	Rats	(99)
	↑ NLRP3 expression; caspase 1/IL-1β activation	Paclitaxel induced CINP	DRG macrophages; DRG and sciatic nerve	Rats	(100)

CFA, complete Freund's adjuvant; CIA, collagen-induced arthritis; CIBP, cancer-induced bone pain; CINP, chemotherapy-induced neuropathic pain; DRG, dorsal root ganglia; FLSs, fibroblast-like synoviocytes; MIA, monosodium iodoacetate; OA, osteoarthritis; RA, rheumatoid arthritis; SNI, spared nerve injury; SNL, spinal nerve ligation. Light grey shading refers to findings in neuropathic models, very light blue in transient inflammatory pain, mid blue in RA and dark blue in OA.

**Table 2:** Overview of mitochondrial dysfunction in patients with rheumatic disease.

Mitochondrial function	Protein/pathway affected	Pathology	Tissue/cell type	References
Mitochondrial respiration	↓ mETC activity and ATP levels	OA	Chondrocytes	(101–103)
	↑ Increased ATPSc-KMT expression	OA	Subchondral bone	(104)
	↓ Expression of mETC complexes	RA, Fibromyalgia	PBMCs	(105–107)
	↓ mETC activity and OxPhos	Fibromyalgia	Skin fibroblasts	(108)
	↓ Expression of OxPhos associated genes	JIA	Peripheral leukocytes	(109)
	↓ ATP levels	SLE	T lymphocytes	(110)
	Complex I deficiency	Muscle Pain	Skin fibroblasts, skeletal muscle	(111)
Oxidative stress	↓ Expression of antioxidant molecules	OA/RA/PsA	Synovium/cartilage	(51, 112–118)
	↑ MtROS	SLE	PBMCs	(119)
	↑ MtROS	Fibromyalgia	Skin fibroblasts	(108)
	↑ MtROS	PsA	Blood monocytes	(118)
Mitophagy	↑ PINK1 expression	OA	Chondrocytes	(44)
Mitochondrial biogenesis	↓ AMPK activity, SIRT-1, PGC-1α, TFAM, and NRF-2 expression	OA	Chondrocytes	(81, 82)
	↓ Expression of PGC-1α, TFAM, NRF1	Fibromyalgia	PBMCs	(105)
Fusion/fission	↑ DRP1 expression	RA	Synovial tissues and FLSs	(120)
NLRP3 inflammasome activation	↑ IL-1β production	OA	Synovial fluid	(91)
	↑ IL-1β and IL-18 production	Fibromyalgia	PBMCs, serum	(105, 108)
	↑ NLRP3 expression	Neuropathic pain	DRG neurons	(121)

FLSs, fibroblast-like synoviocytes; JIA, juvenile idiopathic arthritis; OA, osteoarthritis; PBMCs, peripheral blood mononuclear cells; PsA, psoriatic arthritis; RA, rheumatoid arthritis; SLE, systemic lupus erythematosus.

### Mitochondrial respiration

The five mitochondrial respiratory chain complexes (complex I–V), known as the mitochondrial electron transport chain (mETC), are located in the inner membrane. These series of complexes transfer electrons from nicotinamide adenine dinucleotide (NADH) or flavin adenine dinucleotide (FADH), formed in the tricarboxylic acid cycle (TCA) in the mitochondrial matrix, to oxygen. This electron transfer is needed to create a transmembrane electrochemical gradient by pumping protons across the membrane. The flow of protons back into the matrix through complex V allows ATP production (45, 122, 123). OxPhos is responsible for 90% of the ATP consumed by neurons. Its importance is highlighted by deficits in OxPhos, which reduces dendritic synaptic plasticity in neurons, leading to neuronal injury or even cell death (39, 124). Exposure of neurons to inflammatory cytokines, such as TNF and/or IFNγ, depolarizes the mitochondrial membrane potential, impairs OxPhos and ATP production (125, 126). Conversely, exposure of sensory neurons to IL-17, IL-1α or IL-1β increased mitochondrial respiration to support neurite outgrowth (127, 128). Thus, inflammation affects mitochondrial respiration and neuronal function directly. Importantly, interfering with mitochondrial respiration in neurons affects sensory processing and pain. For example, intrathecal administration of complex I or III inhibitors induced mechanical allodynia in naïve mice (129). Furthermore, complex I deficiency in humans is associated with muscle pain (111). In addition, a deficit in mitochondrial respiration may also indirectly contribute to pain development. Genetic disruption of mETC complex IV activity in mouse DRG neurons increased the adenosine diphosphate (ADP)/ATP ratio due to impaired ATP production. The relative increase in ADP induced mechanical and thermal hypersensitivity through activation of purinergic receptor P2Y1 expressed on sensory neurons afferents (49).

The oxygen consumption rate (OCR), a measure of mitochondrial respiration, is reduced in lumbar DRG neurons at the peak of transient carrageenan-induced inflammatory pain, but is increased again when pain had resolved (25). In CFA-induced persistent inflammatory pain, mass spectrometry analysis identified that several proteins involved in mETC are reduced, suggesting that mitochondrial respiration in the DRG is affected in persistent inflammatory pain (66). In the lumbar spinal cord, OxPhos is reduced in rats with CFA-induced pain, or in mice after chronic constriction induced nerve injury (65), indicating that deficits in mitochondrial respiration may contribute to persistent inflammatory pain and neuropathic pain, respectively. Dichloroacetate (DCA), which activates pyruvate dehydrogenase through inhibiting pyruvate dehydrogenase kinase, boosts the TCA cycle and OxPhos (130). The reversal in mitochondrial respiration deficit induced by DCA in the spinal cord reduced pain-associated behaviors in rats and mice after CFA-induced inflammatory pain and chronic construction induced nerve injury (65). In contrast, when rotenone, a mETC complex I inhibitor that decreases OxPhos, is injected at the site of inflammation, it decreased CFA-induced mechanical hypersensitivity (66). Even though these findings seem contradictory, they may be explained by differential effects of modulating mitochondrial respiration at peripheral nerves and at the spinal cord/DRG. Indeed, preliminary data shows that intrathecal administration of complex III inhibitor myxothiazol into the spinal cord/DRG inhibited hyperalgesic priming, a form of latent nociceptor plasticity, but not when myxothiazol was injected locally in the inflamed paw (64). Moreover, intrathecal injection of rotenone (complex I inhibitor) or antimycin (complex III inhibitor) in naive mice generated persistent pain (129), whilst rotenone decreased CFA-pain when injected into the inflamed hind paw (66). To what extent these differences are merely a difference between non-inflammatory and inflammatory conditions remains to be determined. Moreover these injections do not target only neurons, but also various other cells such as skin cells, immune cells, glia, and others. Thus, differences may depend on which cells are targeted.

There is also evidence that a single inflammatory agent changes mitochondrial activity in sensory neurons. As example, the prototypic inflammatory prostaglandin E_2_ (PGE_2_) increased the TCA cycle and OxPhos through an EPAC2 dependent pathway, contributing to acute inflammatory pain (63). Another indication that mitochondrial respiration may contribute to inflammatory pain is that expression of ATPSc-KMT (also known as FAM173B), a mitochondrial methyltransferase that promotes OxPhos and mtROS production in neurons (131), is increased in DRG neurons of mice with chronic inflammatory pain. Knockdown of ATPSc-KMT in the lumbar DRG during the established chronic inflammatory pain attenuated hyperalgesia. Conversely, expression of ATPSc-KMT in sensory neurons of mice with transient inflammatory pain caused persistent pain (46). These findings may be relevant to rheumatic pain patients because a genetic polymorphism downstream of the ATPSc-KMT gene is linked to joint-specific chronic widespread pain (132). Moreover, ATPSc-KMT expression is increased in the subchondral bone of OA patients and is negatively correlated with pressure pain sensitivity (104). The cells in which ATPs-KMT expression is increased remain to be determined. Nerve cells may be possible candidates, because innervation is increased in the subchondral bone of OA patients (32), but nerve cells are still much less abundant than joint cells in this case.

Hyperalgesic priming triggered by peripheral inflammation affects the plasticity of sensory neurons, by augmenting mRNA translation and causing a switch from cyclic adenosine monophosphate (cAMP) to protein kinase C ε (PKCε-dependent signaling, following a subsequent inflammatory stimulus that acts on G-protein-coupled receptors (GPCRs) (30, 133). Hyperalgesic priming requires the activity of mETC complexes and various mitochondrial proteins are targets of PKCε-dependent signaling (134, 135), pointing to a role of mitochondria in hyperalgesic priming. Activated PKCε promotes the opening of mitochondrial ATP-dependent K^+^ channels (mitoKATP) and increases OxPhos (136, 137). Moreover, preliminary data suggest that hyperalgesic priming increased OxPhos in DRG sensory neurons (64).

Overall, inflammatory mediators alter mitochondrial respiration in neurons. Based on current evidence, some inflammatory agents promote mitochondrial respiration, whilst others reduce it. Possibly, the effect differs depending on the time point after the administration of the inflammatory agent (25). Nevertheless, current data show that both increased and decreased OxPhos result in pain. The question remains why both reduced and increased OxPhos may contribute to pain? Possibly, impaired OxPhos reduces ATP production, which could affect the stability of the membrane potential and disturb neuronal excitability (45, 138). In contrast, increased OxPhos may come at the costs of more production of mtROS as a byproduct of mETC, leading to hyperexcitability, as will be discussed in the next section. Further research will need to contemplate how exactly mitochondrial respiration is linked to sensory neuron function.

### Oxidative stress

Mitochondria generate approximately 90% of cellular ROS (139). MtROS production damages mitochondria in a range of pathologies, including neuropathies (45), and is important in redox signaling (140). Superoxide (O_2_^−^) is the proximal mtROS, mainly released at the mETC during OxPhos. Complex I and Complex III are the major sources of mtROS, and complex II to a lesser extent. Although the mETC is the main source of ROS, several other matrix proteins and complexes, like TCA cycle enzymes (e.g., pyruvate dehydrogenase), or some inner mitochondrial membrane proteins whose activity is partially dependent on mitochondrial membrane potential, also produce ROS.

Approximately 0.2%–2.0% of the oxygen consumed by mitochondria is reduced to O_2_^−^, which is subsequently converted to other ROS, such as hydrogen peroxide (H_2_O_2_) and hydroxyl ions (OH^−^) (140, 141). An imbalance between mtROS production and its removal, either due to ROS overproduction and/or decreased antioxidants defense, causes oxidative stress (139, 142). Neurons, like all other mammalian cells, have antioxidant enzyme systems, such as superoxide dismutase, peroxidases or catalases, which scavenge mtROS when they are generated. Despite contributing to pathology, mtROS also act as signaling molecules, to ensure quality control and maintenance of functional cells, and regulate a variety of physiological processes, such neuronal differentiation, synaptic pruning, and neurotransmission (143, 144). A disturbed mETC is one of the major drivers of mtROS (139), but also decreased mitochondrial membrane potential, disrupted mitochondrial Ca^2+^ buffering, altered mitochondrial morphology, or cellular stress in general augment mtROS production, which disrupts the redox balance and causes oxidative stress that negatively affects neuron function (143, 145).

Neurons are more likely to suffer from mtROS-induced oxidative stress compared to other cell types, because of their large energy consumption, mainly supported through OxPhos. Moreover, neurons have a high content of unsaturated fatty acids and proteins that are vulnerable to oxidation (146). A disrupted redox balance has detrimental consequences for neuronal functioning (139, 141, 147). As an example, mutations in superoxide dismutase (Cu-Zn), also known as superoxide dismutase 1 (SOD1), cause motor neuron degeneration in amyotrophic lateral sclerosis patients (125, 148), of which 60% develop pain (149). Antimycin A (mETC complex III inhibitor) induces mtROS, which activates TRPA1 receptor and increases the excitability of sensory neurons (150–152). Similarly, treatment of spinal cord neurons with ROS donors augmented their excitability and intrathecal administration of these ROS donors induced mechanical hypersensitivity in rats (153). Increased ROS production in the spinal cord also promoted pain through reducing firing of spinal inhibitory neurons. In contrast, others have found that ROS increase the activity of a different subset of inhibitory neurons (154, 155). Although some studies have shown decreased activity, in general, ROS appears to increase neuronal excitability and promote pain.

Both inflammatory and neuropathic pain are associated with increased ROS production in the peripheral and central nervous system. In mice, intraplantar capsaicin administration induced mtROS in spinal cord neurons. Overexpression of the anti-oxidant mitochondrial manganese dependent superoxide dismutase (MnSOD), also called SOD2, prevented capsaicin-induced hyperalgesia, indicating requirement of mtROS for hyperalgesia development (69). Importantly, rare variants present in mETC genes that are major sites of mtROS formation, are associated with the severity of erosive RA (156). Accordingly, increased mtDNA mutations, often a consequence of mtROS, are found in RA and PsA patients (157). These mtDNA mutations affect mitochondrial function and further promote mtROS production (144, 158). Even though these mutations were identified in synoviocytes, similar mutations may occur in sensory neurons innervating chronically inflamed tissues. Indeed, inflammation triggers mtROS production causing mutations in mtDNA in neurons (146). A role for mtROS in pain is further substantiated by findings that overexpression of mitochondrial ATPSc-KMT induced mtROS formation in DRG neurons and prolonged inflammatory pain. The antioxidant phenyl-N-t-butylnitrone (PBN) or the mitochondria-targeted antioxidant mitoTEMPOL reversed the persistent pain induced by ATPSc-KMT overexpression (46, 64). Various other studies showed that antioxidants (e.g., resveratrol) reduced pain in rodent models of OA and neuropathic pain (159–162). Overall, these data indicate that ROS, likely mitochondrial derived, contributes to inflammatory pain.

There is even more evidence for a role of mtROS in neuropathic pain. In spinal dorsal horn neurons, mtROS is increased in rats 1 week after spinal nerve ligation (70). SOD2-like antioxidants reduced mechanical and heat hyperalgesia induced by spinal nerve ligation or chronic constriction injury in rats (163, 164). Chemotherapeutic agents, such as cisplatin and oxaliplatin, cause nerve damage and pain through mitochondrial dysfunction, due to increased mtROS production and decreased antioxidant protection (58, 71, 144, 158, 165, 166). Intraperitoneal administration of mitochondria specific (SS-31, TEMPOL, or a SOD2 mimetic) or unspecific antioxidants (PBN, pioglitazone) reduced ROS production and attenuated pain in chemotherapy-induced neuropathy models (45, 47, 48, 71), showing that mtROS production is a driver of neuronal dysfunction and pain.

Although direct evidence for a contribution of mtROS in the nervous system for pain in rheumatic disease is still lacking, findings in neuropathic pain and inflammatory pain models suggest that oxidative stress induced by mitochondrial dysfunction may contribute to pain by increasing neuronal excitability.

### Ca^2+^ buffering

The endoplasmic reticulum and mitochondria are the two major intracellular Ca^2+^ storages (167). In neurons, mitochondria take up Ca^2+^ into the mitochondrial matrix through the mitochondrial Ca^2+^ uniporter (MCU) complex (55, 167–169). The MCU is Ca^2+^-sensitive and opens by elevated cytosolic Ca^2+^, allowing Ca^2+^ to flow into the matrix. Mitochondria release Ca^2+^ primarily by the Na^+^/Ca^2+^ exchanger (NCLX) (170). The maximal rate of release is much lower than the maximal uptake rate. Therefore, under conditions of continuous high cytosolic Ca^2+^, e.g., due to continuous firing of neurons, mitochondrial Ca^2+^ accumulates. Mitochondria have an enormous capacity to accumulate and store Ca^2+^. In resting neurons, total mitochondrial Ca^2+^ is approximately 100 μM and free mitochondrial Ca^2+^ only 0.1 μM (171). These concentrations steeply increase when neurons are active and mitochondria start accumulating Ca^2+^ (172). In extreme conditions, mitochondrial Ca^2+^ reaches a concentration of up to 1,500 μM (173). In case of mitochondrial Ca^2+^ overload, the mitochondrial permeability transition pore (mPTP) is formed, which releases Ca^2+^ and other molecules, such as Cytochrome C, into the cytosol, inducing apoptosis. This pore has been mainly studied under pathological conditions, and several core components like ATP synthase, cyclophilin D, and the adenine nucleotide translocators are thought to be involved (123, 167, 174).

Only a relatively small fraction of Ca^2+^ is handled by mitochondria during physiological cytosolic Ca^2+^ signals. Nevertheless, mitochondrial Ca^2+^ influx and efflux play a role in the spatiotemporal organization of the cytosolic Ca^2+^ signals (175), which regulates activity-dependent signaling and neuronal excitability in nociceptors, thus is important to prevent aberrant signaling and pain (176). A rise in mitochondrial matrix Ca^2+^ stimulates OxPhos-mediated ATP and mtROS production, regulates organelle dynamics and trafficking, and modulates neurotransmitter release, synaptic transmission, and excitability. Mitochondria are a major regulator of Ca^2+^ signaling at the first sensory synapse (39, 76, 174, 177, 178). Moreover, mitochondrial Ca^2+^ mediates signaling to the nucleus and the release of death signals, in case of very high Ca^2+^ (39, 75, 76, 174, 177, 179–181).

Inflammatory stimuli can lead to a rise in cytosolic Ca^2+^ in sensory neurons, which facilitates the release of neurotransmitters, excitability, and pain (39, 76, 177, 181). Studies showed that inflammatory cytokines increase spontaneous Ca^2+^ oscillations in organotypic spinal cord slices (182). Intraplantar carrageenan or CFA injection in rats increased intracellular Ca^2+^ in brain neurons or increased evoked Ca^2+^ transients in DRG neurons, respectively (72–74). These studies show that cytosolic Ca^2+^ is regulated by inflammation, yet direct evidence that mitochondrial Ca^2+^ is affected during inflammatory pain is lacking. Because mitochondrial and cytosolic Ca^2+^ are interdependent, the data may suggest a potential involvement of mitochondrial Ca^2+^ in inflammatory pain.

Nerve damage causes disturbed mitochondrial Ca^2+^ buffering (180). For example, spinal nerve ligation in rats reduced mitochondrial Ca^2+^ storage capacity in lumbar DRG neurons (75). Rats with chemotherapy-induced neuropathic pain have a decreased duration of depolarization-evoked Ca^2+^ transient, which is partially mediated by an augmented mitochondrial Ca^2+^ uptake and increased mitochondrial volume (78). *In vitro* studies showed that the chemotherapeutic drug paclitaxel induces the formation of mPTP and promotes the release of mitochondrial Ca^2+^, contributing to sensory neuron hyperexcitability and cell death (76). The chemotherapeutic drugs cisplatin and oxaliplatin increased cytosolic Ca^2+^ concentration and depolarization-evoked Ca^2+^ transients in cultured sensory neurons (71, 165). Taken together, different insults disturb distinctive aspects of mitochondrial Ca^2+^ buffering.

Does modulating mitochondrial Ca^2+^ buffering affect neuronal excitability and pain? The noxious heat-activated receptor TRPV1 conducts Ca^2+^ and Na^+^, producing a depolarizing receptor potential that activates nociceptors. Knockdown of NCLX in DRG neurons decreased mitochondrial Ca^2+^ release, reduced capsaicin-induced TRPV1 activation and neuronal firing (183), indicating that mitochondrial Ca^2+^ buffering is important for neurons' ability to respond to pungent reagents. Blocking MCU, with an intrathecal injection of a MCU inhibitor, prevents mitochondrial Ca^2+^ uptake in spinal cord neurons and diminished capsaicin-induced neuron hyperexcitability and pain in mice (184). Mechanistically, by regulating cytosolic and mitochondrial ionic transients, NLCX and MCU modulate Ca^2+^-dependent desensitization of TRPV1 channels, thereby controlling nociceptive signaling (183). Oxaliplatin increases cytosolic Ca^2+^ by increasing the expression of non-selective cation channel TRPV1 in DRG neurons, thus increasing sensory neurons' excitability. Silencing or blocking TRPV1 reduced both oxaliplatin and paclitaxel induced neuropathic pain (185–188), which is indirect evidence that mitochondrial Ca^2+^ buffering is also involved in chemotherapy-induced neuropathic pain. A role for MCU in mechanical allodynia has been found in mice with painful diabetic neuropathy. Selective knockdown of the MCU in Nav1.8-positive DRG neurons resolved mechanical allodynia in diabetic mice (79). Likewise, blocking mPTP through intraperitoneal injection of cyclosporine A reversed spared nerve injury-induced allodynia in rats, by reducing Cytochrome C release and loss in activity of spinal cord GABAergic inhibitory neurons (76, 77).

Mitochondrial Ca^2+^ buffering also plays a role in spinal synaptic plasticity (184). Inhibition of spinal mitochondrial Ca^2+^ uptake in mice, using different pharmacological strategies, blocked N-methyl D-aspartate (NMDA)-induced activation of downstream protein kinases that mediate spinal synaptic plasticity, and reduced induction of long term potentiation, a process that increases synaptic strength in the dorsal horn of the spinal cord and contributes to chronic pain. Importantly, inhibition of mitochondrial Ca^2+^ uptake in the spinal cord prevented animals from developing mechanical hyperalgesia in response to intrathecal NMDA or intradermal capsaicin injection (184).

### Mitochondrial mediated NLRP3 inflammasome activation

Inflammasomes are intracellular multiproteic complexes, composed of a sensor protein that oligomerizes, in order to recruit caspase-1. MtROS production is a main trigger of nucleotide-binding oligomerization domain-like receptor pyrin domain containing 3 (NLRP3) inflammasome activation (189, 190). MtROS causes translocation of the inner mitochondrial membrane protein cardiolipin to the outer mitochondrial membrane, where cardiolipin serves as a docking place for caspase-1 and NLRP3. Subsequently, the adaptor apoptosis-associated speck-like protein containing a CARD (ASC) binds both NLRP3 and caspase-1, activating the inflammasome to initiate caspase-1-mediated cleavage of pro-IL-1β and pro-IL-18 into their active mature form (189). Mitochondria can also sustain NLRP3 inflammasome activation *via* generation of ATP (191) and disturbed Ca^2+^ signaling (189, 190). While NLRP3 inflammasome activation is classically thought to occur in immune cells, such as macrophages, it has also been detected in neurons (97, 192). Moreover, NLRP3 expression is increased in the DRG neurons of patients with neuropathic pain in comparison with controls with no pain (121). Neuronal NLRP3 inflammasome activation likely contributes to neuro-inflammation and neurodegeneration, as it has been detected in brain neurons in Parkinson's and Huntington's disease (193–195). It is not known whether NLRP3 inflammasome activation interferes directly with neuronal functioning.

In the context of rheumatic diseases, mitochondrial dysfunction (e.g., increased mtROS, disturbed OxPhos or Ca^2+^ uptake) may trigger NLRP3 inflammasome activation in the nervous system, affecting sensory processing. For example, in the CFA-induced chronic inflammatory pain, the compound muscone, which diminishes ROS production, prevented the loss of mitochondrial membrane potential and Ca^2+^ influx. Moreover, it blocked the CFA-induced increase in spinal cord NLRP3 and IL-1β expression and hyperalgesia (87, 196). Importantly, the downstream product of inflammasome activation, IL-1β, induces firing of nociceptors and mediates CFA-induced pain (189, 197, 198), suggesting a putative role for NLRP3 inflammasome activation in inflammatory pain.

Although studies about the role of NLRP3 inflammasome in the nervous system in inflammatory pain models are still scarce, various findings suggest the involvement of this pathway in other types of pain. Intraperitoneal or intrathecal administration of MCC950, a specific NLRP3 inflammasome inhibitor, alleviated mechanical allodynia, and decreased IL-1β and IL-18 release in the lumbar dorsal spinal cord in cancer-induced bone pain and oxaliplatin-induced neuropathy, respectively (97, 98). In the cancer-induced bone pain model, IL-1β and IL-18 were detected predominantly in neurons (97). Suppressing NLRP3 inflammasome activation with different microRNAs, reduced NLRP3 inflammasome activation in the sciatic nerve, spinal astrocytes and microglia, while concurrently attenuating mechanical allodynia after chronic constriction injury in mice and rats (92–96). The chemotherapeutic drug bortezomib, which disrupts mitochondrial Ca^2+^ buffering and promotes mtROS production in neurons, increased NLRP3 expression in the DRG (138). Silencing NLRP3 in the DRG prevented the development of bortezomib-induced neuropathic pain in mice and rats (99). Treatment with a ROS scavenger decreased paclitaxel-induced mechanical allodynia and reduced NLRP3 expression in DRG macrophages and caspase 1/IL-1β activation in lumbar DRG and sciatic nerve (100). These studies indicate that nerve damage and inflammation may lead to inflammasome activation in the nervous system, although the majority of these studies did not identify in which cells the inflammasome was activated. Given that nerve damage and inflammation promote mtROS production in neurons, it is possible that mtROS triggers inflammasome activation in neurons and contributes to pain.

### Quality control mechanisms

Mitochondria are highly dynamic organelles and have several quality control pathways in order to maintain their integrity. A controlled balance between mitophagy and mitochondrial biogenesis guarantees optimal mitochondrial turnover. Mitochondrial dynamics involve continuous fission and fusion forming a dynamic network to maintain their content, morphology and quality (145, 199).

#### Mitophagy and mitochondrial biogenesis

Damaged mitochondria are selectively degraded by auto-phagosomes engulfment, followed by lysosomal degradation, a controlled process called mitophagy. Mitophagy involves PTEN-induced kinase 1 (PINK1), which binds the surface of depolarized and damaged mitochondria and to recruit Parkin and trigger mitochondrial aggregation, engulfment, and digestion by lysosomes (44). The production of new functional mitochondria, mitochondrial biogenesis, is mainly driven by peroxisome proliferator-activated receptor-gamma coactivator-1α (PGC-1α). Increased AMP/ATP and ADP/ATP ratios activate the energy sensor AMPK, which activates PGC-1α by phosphorylation. An increase in the NAD^+^/NADH ratio triggers PGC-1α activation *via* sirtuin1 (SIRT1)-mediated deacetylation. Thus, mitochondrial biogenesis is highly influenced by the cellular energy state and redox balance. PGC-1α promotes mtDNA replication and the expression of mitochondrial genes, such as transcription nuclear factors (*NRF-1* and *NRF-2*), which prompt the expression of mitochondrial transcription factor A (TFAM) and mETC complexes subunits (200). Dysfunctional mitophagy or defective mitochondrial biogenesis results in an overall less efficient mitochondrial pool with impaired ATP production and increased mtROS production, which ultimately may affect sensory processing (201).

Some studies suggest a link between dysfunctional mitophagy and pain. Following spinal nerve ligation, PINK1 expression is increased in inhibitory GABAergic interneurons, suggesting increased mitophagy in these spinal cord neurons (80). *Pink1*-KO mice have normal responses to mechanical stimuli, but develop less mechanical allodynia after monosodium iodoacetate (MIA)-induced OA or after spinal nerve ligation (44, 80). Morover, *Pink1*-KO mice developed less spontaneous pain in the second inflammatory phase of formalin-induced pain (202). These data suggest that PINK1/Parkin-mediated mitophagy is required for development of inflammatory, osteoarthritis and neuropathic pain. But why does reducing mitophagy alleviate pain? One could expect that diminished mitophagy would increase the pool of damaged mitochondria, increase mtROS production and enhance neuronal excitability (145). However, in chronic pain states, if the rate of mitophagy is increased whilst not being in balance with the rate of biogenesis, there is an overall depletion of mitochondria. In *Pink1*-KO mice this balance may be restored due to diminished mitophagy (201). Indeed, in conditions such as ischemic stroke, diabetes or autosomal dominant optic atrophy, insufficient biogenesis and excessive mitophagy diminished the number of mitochondria in neurons leading to cell death (203–205). Similarly, decreased mitochondrial biogenesis reduced the total pool of mitochondria in brain neurons in Parkinson's and Alzheimer's disease leading to neuro-inflammation and -degeneration (55, 206, 207).

Some indirect evidence suggests mitochondrial biogenesis is impaired in several pain conditions, because AMPK activation, an essential driver for mitochondrial biogenesis, has an analgesic effect. Local or systemic AMPK activation with various pharmacological compounds reduced formalin, zymosan (208), and CFA-induced inflammatory pain in mice (198, 208). Intrathecal AMPK activation reduced oxaliplatin-induced neuropathy (209), cancer-induced bone pain (210), and painful diabetic neuropathy (211). In addition, systemic AMPK activation using metformin or resveratrol attenuated spinal nerve ligation induced pain (212), post-surgical pain (213, 214), paclitaxel-induced neuropathy (215, 216), and plantar incision-induced hyperalgesic priming (214–216). Notably, AMPK activation reduced the excitability of sensory neurons (209, 212, 217). Unfortunately, effects on mitochondria were not assessed in these studies. Thus, further research is required to confirm that mitochondrial biogenesis is at the root of the analgesic properties of AMPK activation.

#### Fusion and fission

Adjacent mitochondria can merge, a process called fusion, permitting mixture of mitochondrial content. Conversely, fission is when mitochondria constrict and segment. The GTPases mitofusins1 and 2 (MFN1, MFN2) and optic atrophy 1 (OPA1) induce intermembrane fusion, whilst dynamin-related protein (DRP1) drives mitochondrial fission (84, 218). The fission/fusion ratio controls mitochondria's morphology, size and number (219). Damaged mitochondria can undergo fusion, to create a more interconnected and complementary mitochondrial network to reduce cellular stress. Fission is required for mitochondrial biogenesis and promotes mitophagy in case of cellular stress (220). These dynamics are essential for local mitochondrial quality control, which is particularly important in neurons due to their long axons. Excessive fission results in fragmented mitochondria, impaired energy production, and disrupted mitochondrial homeostasis. Fusion, on the other hand, promotes a more efficient and interconnected mitochondrial network (219, 220). Most evidence for a role of these dynamics in neuronal functioning comes from studies in brain neurons. Increased fission diminishes dendritic and axonal branching and contributes to neurodegeneration in an ischemic stroke model (124, 221). Accordingly, inhibition of DRP1 prevents mitochondrial dysfunction, improves neuronal survival and axonal integrity (55, 145, 221, 222). Finally, depletion of DRP1 reduces neurotoxic Aβ oligomers-induced fission and improves mitochondrial health and synaptic activity in cortical neurons, in a mouse model of Alzheimer disease (39, 223). Altogether, excessive fission appears detrimental for neuronal functioning, whilst reducing fission is neuroprotective.

To what extent the balance between fission and fusion controls sensory neuron function has not been extensively explored. Silencing DRP1 or pharmacologically blocking its activity in the spinal cord and in the DRG decreased HIV/AIDS- and chemotherapy-induced neuropathic pain (224). Likewise, intrathecal administration of 2-bromopalmitate, an inhibitor of protein palmitoylation, decreased DRP1 and increased OPA1 expression in spinal cord astrocytes, inhibiting fission and promoting fusion, respectively. Restoring the balance in fission/fusion reduced CFA-induced inflammatory pain and cancer-induced bone pain (83, 84, 225, 226). Intrathecal administration of SRT1720, a SIRT1 agonist, increases SIRT1-mediated mitochondrial biogenesis and decreased spinal cord DRP1 expression, which was associated with a reduction in cancer-induced bone pain and chronic construction injury-induced neuropathic pain (218, 227). The reduction in mitochondrial Ca^2+^ influx in diabetic mice, with a nociceptor-specific deletion of MCU, prevented mitochondrial fragmentation due to excessive fission in DRG neurons, nerve degeneration, and pain (79). Additionally, intrathecal injection of resveratrol to reduce spinal cord oxidative stress, decreased spinal DRP1 activity and reversed cancer-induced bone pain in rats (159). These data clearly show that mitochondrial dynamics are linked to various other mitochondrial functions and to sensory processing. Inflammation disturbs OxPhos, promotes oxidative stress and Ca^2+^ influx into the mitochondria *via* MCU. All these processes can induce mitochondrial fission (127, 128, 182, 228). As such, inflammation is more likely to induce mitochondrial fission rather than fusion (229), but future research is needed to fully understand how fission and fusion contribute to pain in rheumatic disease.

### Mitochondrial transport

Mitochondrial biogenesis occurs close to the nucleus, since the majority of mitochondrial proteins are encoded by the nuclear genome. In neurons, mitochondria are mainly made in the soma, therefore mitochondria have to be transported to the dendrites and axonal terminals (230). Some studies identified that mitochondrial biogenesis also occurs to some extent in the axons, but the mechanisms are not completely clear (200). Motor proteins from the kinesin family mediate anterograde transport of new mitochondria from the soma to the axons, whilst motor proteins that form a dynein-dynactin complex mediate retrograde transport towards the soma (199, 230). Motor proteins interact with the microtubule network and mitochondrial Rho GTPases (MIROs) to regulate mitochondrial motility for both anterograde and retrograde transport (231).

Neuron-specific knockdown of *Miro1* in mice depletes mitochondria in dendrites and diminishes neuron survival, highlighting the importance of mitochondrial motility for neuronal homeostasis (232). Impairing mitochondrial transport by decreasing the activity of motor proteins in cervical ganglion neurons diminished the number of axonal and dendritic mitochondria, which was associated with synaptic dysfunction that limited neurotransmission and neuroplasticity (231). Restoring the diminished local ATP pool that was affected by the impaired transport, reestablished these parameters, indicating that mitochondrial transport is essential to maintain sufficient ATP levels throughout neurons (233). Enhancing mitochondrial transport in adult murine DRG or cortical neurons by knockdown of syntaphilin, an anchoring protein that keeps mitochondria static, improved axonal regrowth and normalized cellular ATP/ADP ratio after axotomy (233, 234). Inflammation in the peripheral nervous system, induced by experimental autoimmune neuritis, reduced retro- and anterograde transport of mitochondria in nerves (126), indicating that inflammation reduces mitochondrial transport. Similarly, exposure of murine brain slices to lipopolysacharide (LPS) reduced retrograde transport (235). Thus, efficient mitochondrial transport is a requirement for effective energy production and synaptic transmission in neurons. Inflammation can reduce this mitochondrial transport through axons, thereby affecting the local ATP pool.

Histone deacetylase 6 (HDAC6) deacetylates the cytoskeletal protein α-tubulin, limiting mitochondrial transport and distribution in neurons. Systemic administration of HDAC6 inhibitors decreased inflammation-induced mechanical hypersensitivity in CFA- and collagen-induced arthritis models (236, 237), and neuropathic pain models (236, 238). Pharmacological inhibition of HDAC6 also decreased LPS-induced neuronal loss and pro-inflammatory cytokines production in the brain, demonstrating a neuroprotective effect of improving mitochondrial transport (239, 240). Cognitive impairment and neuro-inflammation (microgliosis, production of TNF and IL-1β) in the spinal cord/brain in neuropathic pain models are reversed by HDAC6 inhibition (236, 238). In line with the previous results, oral administration of HDAC6 inhibitors, *Hdac6* knockout, or cell-specific deletion of *Hdac6* in advillin-positive sensory neurons, promoted mitochondrial transport in sensory neurons, attenuated mechanical hyperalgesia, and spontaneous pain in chemotherapy-induced neuropathy (85, 86). HDAC6 inhibition reestablished OCR in lumbar DRG neurons and the tibial nerve of cisplatin-treated mice to the values of control animals (85, 86), indicating mitochondrial transport in neurons is essential to ensure sufficient OxPhos when damaged by neurotoxic agents. Currently, oral administration of ACY-1215 (Ricolinostat, HDAC6 inhibitor) is being assessed in patients suffering from painful diabetic peripheral neuropathy (clinical trial NCT03176472). A selective HDAC6 inhibitor reduced the inflammatory phenotype of immune cells from RA patients *in vitro* and pre-clinical studies show an analgesic effect of this approach in RA models (236, 237, 241). Thus facilitating mitochondrial transport through HDAC6 inhibitors may be a promising approach to relieve rheumatic pain.

## Non-neuronal mitochondrial alterations as cause of pain in rheumatic diseases

Mitochondrial defects in non-neuronal cells may also contribute to changes in sensory processing. Neuro-inflammation contributes to inflammatory and neuropathic pain (65, 92, 95, 210, 238, 242, 243). Mitochondrial dysfunction is a driver of neuro-inflammation and neuronal damage in neurodegenerative diseases (34, 229). Impaired mitochondrial function, such as disturbed Ca^2+^ buffering or increased oxidative stress, in neurons may result in the intracellular release of mitochondrial components, such as mitochondrial DNA, mtROS or Cytochrome C. When extracellular, these components act as damage-associated molecular patterns (DAMPs) that can trigger an inflammatory response and engage spinal microglia or DRG macrophages (34). On the other hand, inflammatory mediators released by the immune cells can contribute to mitochondrial dysfunction, further promoting neuro-inflammation (34, 229). Tissue damage and inflammatory processes in rheumatic joints induce mitochondrial alterations in cells of the joint, such as chondrocytes and synoviocytes. These mitochondrial defects may promote the release of inflammatory mediators that regulate neuronal activity and pain.

### Mitochondrial respiration, oxidative stress and senescence

In several models of rheumatic diseases and in rheumatic patients, mitochondrial respiration is reduced in cells of the joint. As an example, chondrocytes of rabbits and guinea pigs with experimental OA, have an impaired mitochondrial respiration, reduced intracellular ATP levels, and a rise in the lactate/pyruvate ratio (67, 68). These differences worsened during the progression of cartilage damage (68). Similarly, chondrocytes collected from OA patients have decreased activity of mETC complexes and reduced ATP levels compared to control patients, which correlated with an increase in the number of apoptotic chondrocytes (101–103). Apoptotic chondrocytes release substances that can activate receptors on sensory neurons and elicit pain (244). Increased formation of oxidative metabolites (e.g., H_2_O_2_) and decreased expression of endogenous antioxidant molecules (e.g., SOD2, peroxidase) are found in the knee (synovium and cartilage) in pre-clinical rheumatic disease models (112, 245), and in humans with OA (112–116), RA (51, 115, 117) and PsA (118). In RA, an imbalance between oxidants and antioxidants leads to mitochondrial oxidative stress in synoviocytes, which triggers activation of transcription factor NF-κB and subsequent release of inflammatory mediators, such as IL-8 or PGE_2_ (246), that can activate receptors on sensory neurons to induce pain (247, 248). MtROS is important in this process, because mitoTEMPOL, a mitochondrial specific ROS scavenger, reduced the release of inflammatory mediators by human synoviocytes (246).

Oxidative stress can also induce apoptosis of chondrocytes *via* reduction in the expression of FOXO3, a transcription factor upstream of various antioxidant genes, such as *SOD2* (248, 249). Intra-articular or oral treatment with antioxidants, such as amobarbital, N-acetylcysteine (NAC), resveratrol or methylene blue have analgesic effects in different species (mice, rat, porcine) with OA (250, 251) and CFA-induced joint inflammation (160). Similarly, a specific mitochondrial targeted-antioxidant, plastoquinonyl-decyl-triphenylphosphonium bromide (SkQ1), reduced bone destruction and cartilage damage in OA mice (252). Chondrocytes damage may lead to the release of neurotrophin NGF by immune cells, which activates its high-affinity receptor TrkA on neurons and induces expression of a variety of ion channels (e.g., TRPV1, voltage gated sodium channels) and pain (253, 254).

Increased ROS production is a feature of senescence cells, i.e., cells that have entered a non-proliferative state. Cellular senescence is accompanied by a distinct secretory phenotype, senescence-associated secretory phenotype (SASP), which includes a variety of secreted proteins, cytokines and chemokines that can drive chronic inflammation (255, 256). Recently, cellular senescence has been identified in nervous tissue in mice with long-lasting (>4 months) neuropathic pain. Intriguingly, p53-mediated senescence drove neuropathic pain specifically in male, but not female mice. Moreover, a mutation in the *P53* gene is associated with chronic pain in men (256). In mice with experimental OA, the number of senescent cells in the cartilage is increased. Selective elimination of these cells, either genetically or pharmacologically by intra-articular administration of a senolytic, reduced pain-associated behavior and enhanced cartilage reconstruction (257). Similarly, blocking p53-mediated senescence or removing senescent cells reversed neuropathic pain, by reducing the expression of SASP effectors (e.g., p53, IL-1β, IL-6) (256, 258). These results indicate a potential role of mitochondria-driven cellular senescence in pain.

### Mitochondrial mediated NLRP3 inflammasome activation

NLRP3 inflammasome activation has been linked to amplified inflammatory cytokine signaling in senescent cells (259, 260). The expression of NLRP3 inflammasome related molecules (NLRP3, ASC, caspase-1, IL-1β, IL-18) is increased in the synovial tissue of collagen-induced arthritis (CIA) mice (88, 192, 261) and MIA-induced OA rats (89, 90). Intraperitoneal administration of the specific NLRP3 inhibitor MCC950, or blocking IL-18, diminished synovial inflammation and cartilage damage in RA. Moreover, pharmacological inhibition of the inflammasome products IL-1β or IL-18 attenuates RA or neuropathic pain (262, 263). Additionally, administration of NLRP3 inflammasome inhibitors reversed NLRP3 activation in FLSs and relieved OA-induced pain (89, 90). Moreover, indirect regulation of the inflammasome, by downregulating microRNA miR-30b-5p in the joint, attenuated the increase in NLRP3, ASC, and cleaved caspase-1 in joints, and decreased cartilage damage and pain in OA rats (91). In the joint tissue of OA patients, miR-30b-5p is elevated compared to healthy controls and its expression correlates with disease severity and levels of IL-1β in the synovial fluid (91), suggesting a putative role of inflammasome activation in OA patients. However, it remains unclear if inflammasome activation also mediates pain in this rheumatic disease in humans, as pain-associated outcomes were not investigated in this study.

Interestingly, the expression of translocator protein (TSPO), found in the outer mitochondrial membrane, is increased in the synovial membrane of OA patients and its expression levels correlated with lower pain intensity (104). Although TSPO has been commonly used as a target for PET imaging to assess neuro-inflammation and microglia/macrophage activation (264), it is a mitochondrial protein that has also been linked to mitochondrial bioenergetics. Knockout of TSPO in human microglia cell lines reduced OxPhos and ATP production. In addition, TSPO ligands in *in vivo* models prevented mitochondrial membrane depolarization, mPTP formation and NLRP3 inflammasome activation (265, 266), suggesting TSPO activity may indirectly affect some mitochondrial functions. Thus, the TSPO correlation with lower pain intensity may potentially link to overall better mitochondrial performance.

### Quality control mechanisms

Mitochondrial quality control mechanisms are essential to maintain cellular homeostasis and prevent the production of inflammatory cytokines. Intra-articular MIA administration, to induce experimental OA in rats, increased the expression of PINK1 and Parkin in knee cartilage. Likewise, treatment of human chondrocytes with MIA increases PINK1 expression in these cells. In OA patients, PINK1 expression in chondrocytes in damaged parts of joint cartilage is higher compared to healthy cartilage (44). These data point to exacerbated PINK-1-mediated mitophagy in chondrocytes, which may result in cartilage damage and pain. Several studies also described possible disturbances in mitochondrial biogenesis in chondrocytes of rodents or OA patients, since AMPK activity, SIRT-1, PGC-1α, TFAM, and NRF-2 expression are reduced compared to controls (81, 82). In CIA-induced arthritis, intraperitoneal administration of mitochondrial fission inhibitor mdivi-1 reduced arthritis scores and paw thickness in mice (120). Moreover, DRP1, a protein that promotes fission, is increased in synovial tissues and FLSs from RA patients and in primary human chondrocytes following MIA treatment (120). In RA, DRP1 expression correlates with disease severity (44). Finally, neurotrophins, such as NGF or BDNF, are released in the synovial fluid of RA, OA and AS patients and can induce mitochondrial fission in neurons (267–269). These data suggest that mitochondrial fission is enhanced in various rheumatic diseases and that inhibiting fission reduces arthritis. Whether changes in fission also contribute to pain remains to be explored.

In conclusion, several mitochondrial functions are clearly affected in the local tissue in rheumatic diseases. It is not fully understood how these mitochondrial alterations contribute to rheumatic pain. We propose that changes in mitochondrial functions in the site of injury may promote the release of inflammatory mediators that sensitize or activate the nervous system, contributing to pain.

## Systemic and genetic links to mitochondrial alterations in rheumatic diseases

Collection of human joint tissue is relatively common in rheumatic conditions, because it can be obtained during total knee or hip replacement surgeries. On the other hand, DRG, nerves and spinal cord are not easily accessible tissues, which explains the lack of human data regarding mitochondrial functions in the nervous system in rheumatic disease. In contrast, blood is very easily accessible and there is a correlation between mitochondrial dysfunction in blood cells and several other tissues (270, 271). Moreover, studies suggest that mitochondrial alterations in blood cells could be used as a peripheral marker for diseases affecting the nervous system (272, 273). For these reasons, there are several studies that have explored changes in mitochondrial functions in blood components and cells from patients with rheumatic diseases, which could shed some light into how mitochondrial alterations may be associated with rheumatic conditions. An overview of the findings in human patients is depicted in Table 2.

Compared to healthy controls, peripheral blood mononuclear cells (PBMCs) from RA and fibromyalgia patients express less mETC complexes at the protein level, have reduced cellular ATP levels, and have a diminished mitochondrial membrane potential (105–107). Similarly, genes associated with OxPhos are downregulated in peripheral leukocytes of JIA patients in comparison with healthy children. In SLE patients, ATP levels are reduced in T lymphocytes (109, 110). PBMCs and skin fibroblasts from patients suffering from SLE (119) and fibromyalgia (108), respectively, have elevated mtROS levels, similar to what was observed in blood monocytes from PsA patients (118). Given that all these rheumatic diseases have an inflammatory nature, and that inflammatory mediators affect mitochondrial functions and cellular metabolism in immune cells (274, 275), it is highly possible that these changes are caused by inflammation. Conceivably, similar changes are induced in sensory neurons and affect their function.

In fibromyalgia patients with pain, complex III activity, the expression of multiple mitochondrial biogenesis related genes (*PGC-1α*, *TFAM*, *NRF1*), and the expression of coenzyme Q10 are decreased in PBMCs and skin fibroblasts. In contrast, mtROS production is increased compared to controls without pain (105). These data suggest that mitochondrial dysfunction in PBMCs is linked to pain in fibromyalgia. Moreover, inflammasome related molecules (e.g., IL-1β, IL-18) were significantly increased in serum of fibromyalgia patients and correlated with pain VAS scores (108). In RA, mutations in mitochondrial related genes are detected in *MT-ND1*, which encodes a complex I subunit, as well as in *NLRP3* and *CARD8*, which recruits caspase to form NLRP3 complex (276). Additionally, a mutation in the genomic region encoding ATPSc-KMT, a mitochondrial localized protein, is linked to chronic widespread pain and increased pressure pain sensitivity in OA (104, 132).

In conclusion, these data suggest that markers of mitochondrial dysfunction and mitochondrial related genetic modifications are present in rheumatic patients. In some cases, these mitochondrial alterations are associated with the magnitude of pain. Future research has to show whether these cause pain, or are rather a consequence of the pathology that is causing pain.

## Targeting mitochondrial function: a promising approach for chronic pain treatment?

Over the recent years, the possibility to target mitochondria for the treatment of several pathologies, like primary mitochondrial diseases, neurodegenerative diseases, heart failure, chronic kidney disease, and stroke-like episodes has gained attention (277, 278). Even though most evidence for efficacy comes from pre-clinical work, some compounds that target mitochondria are already being assessed for safety in humans. Improving mitochondrial bioenergetics by targeting deficits in mitochondria is still at an early development stage. We will discuss various compounds that are being developed and could be of interest for treating painful (rheumatic) diseases.

### Mitochondrial respiration

Given that inflammation reduces mitochondrial respiration in sensory neurons and diminished OxPhos is associated with prolonged pain, boosting mitochondrial respiration may resolve pain. DCA boosts the TCA cycle and OxPhos. DCA reduced pain-associated behaviors in pre-clinical inflammatory pain models and showed a safe profile when tested in humans (65, 130, 279). DCA reduced lactate levels in endometriosis cells, and is currently being tested as treatment for endometriosis-associated pain (clinical trial NCT04046081) (279).

Altered mitochondrial respiration can lead to unfavorable cellular redox balance, e.g., NAD/NADH ratio. NAD^+^ is an essential cofactor in the regulation of mitochondrial health. A decline in NAD^+^ in various tissues and cells, including neurons, is associated with pathologies, such as neurodegenerative diseases, diabetic-induced and chemotherapy-induced neuropathic pain (280–283). Recently, preliminary data showed that peripheral inflammation reduces NAD^+^ levels in DRG of mice. Systemic or intrathecal administration of nicotinamide riboside, a precursor of NAD^+^, reversed CFA-induced inflammatory pain (64). Importantly, NAD^+^ supplementation with NAD^+^ precursors, such as nicotinamide riboside, mitigates oxidative stress and improves mitochondrial functions. More than 70 years ago, studies already showed that NAD^+^ supplementation reduces pain in RA patients (284, 285). More recently, oral NAD^+^ supplementation reversed diabetic and chemotherapy-induced neuropathic pain in pre-clinical models (281, 283). Other recent studies also showed that NAD^+^ supplementation has favorable outcomes on RA and SLE, but pain was not assessed in these studies (286). NAD^+^ supplementation also improved the global impact of OA, possibly through the increase of NAD^+^ in the synovial fluid and cartilage matrix, ensuring proper energy levels for cartilage repair. However, in this study NAD^+^ supplementation did not affect pain (287). Recent clinical trials in larger cohorts and with more extensive assessment exams have confirmed that oral supplementation with NAD^+^ precursors (including nicotinamide riboside) are safe and well-tolerated (288, 289). Future larger studies will have to show whether NAD^+^ supplementation may hold promise for treatment of inflammation and pain in patients with rheumatic disease.

### Oxidative stress

Antioxidants are promising in reversing oxidative stress in diseases of the nervous system (290, 291). The antioxidant resveratrol is effective in managing rheumatoid arthritis by decreasing plasma inflammatory markers and joint swelling, when given in combination with common anti-rheumatic drugs (117). Similarly, resveratrol administered as adjuvant with the anti-rheumatic drug meloxicam decreased serum levels of inflammatory mediators (TNF, IL-1β, IL-6) and reduced pain by ∼70%, compared to OA patients that received meloxicam together with placebo (292, 293). Oral resveratrol also attenuated chronic musculoskeletal pain by 20% in postmenopausal women (294), indicating a potential analgesic effect of resveratrol in several pain conditions. Currently studies are further exploring resveratrol's analgesic effect in knee OA patients as combined (anti-inflammatory drugs and/or analgesics) and as mono-therapy (clinical trial NCT02905799) (295).

### NLRP3 inflammasome

Pre-clinical studies show that targeting inflammasome-related proteins has analgesic effects (92–98). In a clinical trial, oral treatment with Dapansutrile, a specific NLRP3 inflammasome inhibitor, reduced joint pain 56%–68% and reduced joint and systemic inflammation in gout flare patients (296). Other drugs that target the inflammasome pathway, such as the caspase-1 inhibitor Pralnacasan, the anti-IL18 monoclonal antibody GSK1070806, and compounds targeting IL-1β or its receptor (e.g., anakinra and canakinumab), are being clinically evaluated or already approved to treat inflammatory diseases, including rheumatic disease. Anakinra and canakinumab reduce pain in RA and JIA, but they still need to be assessed in other rheumatic conditions. The ability of GSK1070806 to reduce pain has not been studied (clinical trial NCT03681067) (297–300).

### Mitochondrial transfer/transplantation

Mitochondria are transferred between cells *via* extracellular vesicles, tunneling nanotubes, gap junctions or free mitochondrial ejection (42, 301–308). Intercellular transfer of healthy mitochondria can improve the energy production status of recipient cells and restore their viability. The release of damaged mitochondria might function as a help request to surrounding cells, triggering signaling pathways to restore homeostasis in the cell releasing damaged mitochondria (42, 301–304). Mitochondrial transfer has been observed in a growing list of pathologies that include brain injury (42, 309), neurodegenerative diseases (306, 310), cancer (303, 311, 312), and cardiomyopathy (304). Mitochondria can be transferred from human synovial mesenchymal stem cells (sMSCs) to Th17 cells, which reduces the production of the pro-inflammatory cytokine IL-17. Mitochondrial transfer between sMSCs and Th17 is impaired in RA patients (313). Likewise, sMSCs donate mitochondria to stressed articular chondrocytes and failure of this transfer has been hypothesized to impair healing of orthopedic tissues (314). Importantly, transfer of mitochondria from macrophages to DRG neurons is required to resolve inflammatory pain (25). These findings highlight that intercellular mitochondrial transfer may be an approach to reduce inflammatory pain.

Some research groups have explored the delivery of entire functional mitochondria into damaged cells, a technique called mitochondrial transplantation (315, 316). Pre-clinical studies show mitochondrial transplantation is neuroprotective (317, 318). Additionally, a small clinical trial with 5 patients showed that autologous mitochondrial transplantation (i.e., mitochondria from non-ischemic skeletal muscle of each patient were injected in the myocardium) improved myocardial function in patients with ischemia-reperfusion injury, without inducing short-term complications (319). In all these studies, “naked”/free mitochondria were directly injected in the tissue of interest. Classically, some free mitochondria components are thought to act as DAMPs, thus triggering an inflammatory response (317). However, “naked” mitochondria are found in the human blood, suggesting that healthy free mitochondria are not necessarily inflammatory (320). Nevertheless, to prevent any adverse effect of free mitochondria, packaging mitochondria into vesicles would prevent them from acting as DAMPs, whilst it would allow specific targeting strategies by adjusting lipid composition. Synaptosomes are lipidic membranous particles released at nerve terminals. Synaptosomes can be obtained by homogenization and gradient centrifugation of nervous tissue (synaptic terminals), and contain several synaptic vesicles and mitochondria. Synaptosomes are specifically taken up by neuronal cells, making them an ideal delivery system to target neurons (316).

In summary, targeting mitochondria shows some beneficial effects in multiple pathologies and could be a novel approach to treat chronic pain. The challenge is to develop a strategy to deliver a drug to mitochondria in specific cell types. Possibly, encapsulation of drugs/mitochondria into nanolipidic carriers may overcome these challenges (321–324) and allow the use of mitochondria/mitochondria targeting drugs to treat pain. As an example, in a pre-clinical study, extracellular vesicle containing mitochondria resolved persistent inflammatory pain, albeit transiently (25).

## Conclusion and future perspectives

**Figure 2:**
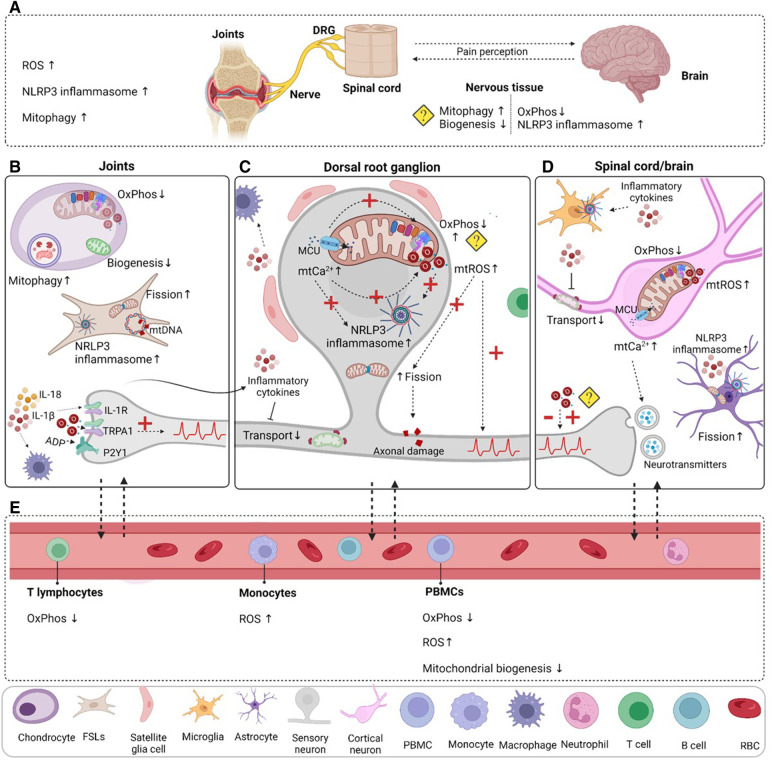
Mitochondrial dysfunction in rheumatic pain conditions. (**A**) Oxidative stress, NLRP3 inflammasome activation and mitophagy are increased in the joint in various rheumatic conditions. Mitochondrial dysfunction is not limited to the damaged joint tissue; it is also present in the nervous system. Decreased mitochondrial respiration and increased inflammasome activation have been detected in RA animal models. Indirect evidence points to decreased mitochondrial biogenesis and excessive mitophagy in the nervous system, but it is not clear in which cells. (**B**) Evidence from clinical and pre-clinical studies points to impaired mitochondrial functions in chondrocytes and fibroblast-like synoviocytes (FLSs), which could contribute to the inflammatory milieu observed in the joint and may activate resident immune cells like macrophages. Mutations in mtDNA may contribute to impaired mitochondrial function. Peripheral inflammation and damage activate and induce long-lasting changes in sensory neurons, and increase their excitability. (**C**) Sensory neuron activation increases mitochondrial Ca^2+^ levels that promote OxPhos, but also mtROS production. Continuous mtROS production may impair mitochondrial respiration or cause mtDNA mutations. Inflammation and oxidative stress reduce mitochondrial transport and impair quality control mechanisms (mitophagy, mitochondrial biogenesis, fusion and fission). Disturbed mitochondrial Ca^2+^ buffering and mtROS trigger NLRP3 inflammasome activation in neurons. (**D**) Prolonged activation of nociceptors in the periphery affects the spinal cord/brain axis of pain processing and can induce permanent central changes in neuroplasticity and mitochondrial dysfunction in neurons and supporting cells like astrocytes. (**E**) Changes in mitochondrial functions in blood components and cells appear to be present in patients with various rheumatic diseases. Even if these alterations were not directly linked to pain, they could shed some light on how mitochondria are affected in rheumatic disease. A question mark is used when there is indirect evidence or there are contradictory findings. Dashed arrows were used to represent hypothetical connections or consequences that have not been directly linked to rheumatic pain. DRG, dorsal root ganglia; PBMC, peripheral blood cells; RBC, red blood cell. Figure created with BioRender.com.

The contribution of mitochondrial dysfunction for chemotherapy, diabetes and HIV-induced neuropathic pain is clear and has been extensively reviewed elsewhere (45). Currently available data point to changes in mitochondria in various cells/tissues in rheumatic diseases (Figure 2; Tables 1, 2). In rheumatic diseases, peripheral inflammation at the joint level may affect mitochondrial function, and mitochondrial dysfunction can further promote inflammation. These inflammatory mediators may affect mitochondria function in sensory neurons, impacting their excitability. Pain development has been associated with altered OxPhos, disturbed Ca^2+^ buffering, increased mtROS production, NLRP3 inflammasome activation and defective mitochondrial quality control mechanisms in the nervous system, but also surrounding cells, such as astrocytes, microglia or other immune cells. Various mitochondrial functions in sensory neurons have been linked to changes in sensory processing, but it is important to note that mitochondrial functions are tightly connected. Therefore, it remains difficult to know if a specific function controls pain/sensory processing. Finally, given the intertwined role of damage and inflammation in mitochondrial function and vice versa, it remains difficult to conclude if mitochondrial dysfunction is a cause or a consequence of rheumatic diseases.

A striking observation is that many of the pre-clinical studies investigating mitochondria in pain used only male animals. Given that RA, OA, SLE or fibromyalgia are more prevalent in females than males (325–328) and various studies show that pain mechanisms are sex-dependent (256, 329–332), sex differences should be taken into account in the contribution of mitochondria to sensory processing. Especially since mitochondrial biogenesis and mitochondrial-mediated cell death signaling after oxygen and glucose withdrawal are sex dependent (333, 334).

Various alterations in mitochondrial functions, such as respiration, oxidative stress, biogenesis and NLRP3 inflammasome activation appear to occur both in human and mice in the context of rheumatic disease. Other studies have also shown similarities between rodents and humans, e.g., they have a similar distribution of synaptic and non-synaptic mitochondria in the brain (335). Moreover, mitochondrial response to aging is conserved between different species (humans, monkey, mice, rats) in terms of changes in mETC, OxPhos, oxidative stress and mitophagy at the pathway level (336, 337). Nevertheless, there are examples of species differences. For example human and mouse astrocytes have different OxPhos rates *in vitro*, with human astrocytes being more susceptible to oxidative stress (338). Regardless, to gain an accurate understanding of the putative role of mitochondria in pain, more research should include humans material, as findings in rodents, the most commonly used animals in pain research, are not always translatable to humans (339). A limitation of the rodent models is that behavioral assays used often do not assess actual pain, but rather pain-associated behavior such as hyperalgesia. As such, most existing data only shows a relation with the development of hypersensivity to noxious stimuli and not *per se* spontaneous pain (340). At the molecular level, species difference may also exist. Even though the overall signature of DRG-enriched genes is conserved between mice and human (341), various ion channels or cholinergic receptors are differentially expressed in neuronal subpopulations between mouse versus human DRG (342). Finally. aging is a risk factor for rheumatic diseases and patients experience pain during years (340, 343). However, for ethical and financial reasons, pain and putative underlying mechanisms are rarely followed for more than a few months in animal models. More studies should be performed in aged mice and at later time points after the model is established (256, 340), because potentially relevant aspects are missed. As example, targeting senescence, which is closely linked to mitochondrial function, in the spinal cord decreased neuropathic pain when it was established for 9 months, but not when it was only established for 2 weeks after spared nerve injury (199, 256). These rodent models are still valuable, because some compounds that had an analgesic effect in pre-clinical studies (e.g., NAD^+^ supplementation, resveratrol, NLRP3 inflammasome inhibitors) also relieved pain in clinical trials (64, 97, 98, 160, 161, 281, 283, 285, 292, 293, 298, 299).

Until now, human data on mitochondria function in the nervous system is scarce. Skin biopsies and, occasionally, DRG can be collected from live human donors, but central nervous tissues are usually obtained only *post mortem* (339). Interestingly, recent imaging techniques may allow to obtain this information to a certain extent. Imaging techniques (e.g., magnetic resonance spectroscopy, infrared spectroscopy or positron emission tomography) can be used to measure mitochondrial oxygen consumption, mitochondrial membrane potential, ATP and metabolite levels in the brain or spinal cord (278, 344). For example, TSPO-PET, which has been commonly used to measure immune cell activation in the nervous system, may also be useful to assess mitochondrial respiration and function, according to some recent pre-clinical studies (265, 266, 345). Therefore, these non-invasive techniques could potentially be applied to collect information about mitochondrial dysfunction not only in the brain, but also DRG, spinal cord and nerves in rheumatic pain patients.

In conclusion, impaired mitochondrial health at the peripheral and central level may contribute to rheumatic pain. Considering the central role of mitochondria in cellular function after inflammation and their emerging role in sensory processing, modulation of mitochondrial functions may be a promising approach to attenuate or eliminate pain in rheumatic diseases.
